# Determining the Best Noninvasive Test for Peripheral Arterial Disease Diagnosis to Predict Diabetic Foot Ulcer Healing in Patients Following Endovascular Revascularization

**DOI:** 10.3390/healthcare12161664

**Published:** 2024-08-20

**Authors:** Francisco Javier Álvaro-Afonso, Yolanda García-Álvarez, Esther Alicia García-Morales, Sebastián Flores-Escobar, Luis De Benito-Fernández, Jesús Alfayate-García, Juan Pedro Sánchez-Ríos, Enrique Puras-Mallagray, Esteban Javier Malo-Benages, Marta Ramírez-Ortega, Sandra Redondo-López, Almudena Cecilia-Matilla, José Luis Lázaro-Martínez

**Affiliations:** 1 Diabetic Foot Unit, Clínica Universitaria de Podología, Facultad de Enfermería, Fisioterapia y Podología, Universidad Complutense de Madrid, Instituto de Investigación Sanitaria del Hospital Clínico San Carlos (IdISSC), 28040 Madrid, Spain; alvaro@ucm.es (F.J.Á.-A.); eagarcia@ucm.es (E.A.G.-M.); jhflores@ucm.es (S.F.-E.); diabetes@ucm.es (J.L.L.-M.); 2 Diabetic Foot Unit, Angiology and Vascular Department, Hospital Universitario Fundación Alcorcón, 28922 Alcorcon, Spain; luisde.benito@salud.madrid.org (L.D.B.-F.); jesusmanuel.alfayate@salud.madrid.org (J.A.-G.); jsrios@salud.madrid.org (J.P.S.-R.); 3 Angiology, Vascular & Endovascular Department, Hospital Universitario Quirónsalud Madrid, 28223 Pozuelo de Alarcon, Spain; enrique.puras@quironsalud.es (E.P.-M.); esteban.malo@quironsalud.es (E.J.M.-B.); 4 Angiology, Vascular & Endovascular Department, Hospital Universitario La Luz, Grupo Quironsalud, 28003 Madrid, Spain; marta.ramirezo@quironsalud.es; 5 Vascular Surgery Service, Ruber International Hospital Madrid, c/Masó 38, 28034 Madrid, Spain; sandraredondolopez@gmail.com; 6 Diabetic Foot Unit, Vascular Surgery Service, Hospital Universitario Ramón y Cajal, Instituto Ramón y Cajal de Investigación Sanitaria (IRYCIS) Crta, Colmenar Viejo Km 9100, 28034 Madrid, Spain; almudena.cecilia@salud.madrid.org

**Keywords:** diabetic foot, diabetic foot ulcer, diagnosis, peripheral arterial disease, endovascular revascularization

## Abstract

Background/Objectives: To analyze the best noninvasive tests prognosis marker in patients with diabetic foot ulcer (DFU) who underwent endovascular revascularization based on clinical outcomes, such as healing rate, time to heal, and free amputation survival after at least a six-month follow-up. Methods: A multicentric prospective observational study was performed with 28 participants with ischemic or neuroischemic DFU who came to the participant centers and underwent endovascular revascularization between January 2022 and March 2023. Toe systolic pressure (TP), ankle systolic pressure (AP), the ankle brachial pressure index (ABPI), the toe brachial pressure index (TBPI), transcutaneous pressure of oxygen (TcPO_2_), and skin perfusion pressure (SPP) were evaluated using PeriFlux 6000 System, Perimed, Sweden, before (Visit 0) and four weeks after revascularization (Visit 1). The primary clinical outcome was an evaluation of the clinical evolution of noninvasive tests comparing Visit 0 and Visit 1, estimating the sensitivity for predicting wound healing of noninvasive tests at six months following initial recruitment. Results: After six months, 71.43% (n = 20) of DFU healed, four patients (14.3%) received major amputations, and one (3.5%) died. The two tests that best predicted wound healing after revascularization according to the ROC curve were TcPO_2_ and TP with sensitivities of 0.89 and 0.70 for the cut-off points of 24 mmHg and 46 mmHg, respectively. Conclusions: TcPO_2_ and TP were the two tests that best predicted wound healing in patients who underwent endovascular revascularization. Clinicians should consider the importance of the evaluation of microcirculation in the healing prognosis of patients with diabetic foot ulcers.

## 1. Introduction

Diabetic foot ulcer (DFU) is one of the most common complications of diabetes mellitus. The lifetime incidence of DFU in patients with diabetes is approximately 19% to 34% [1] and the recurrence rate is estimated at 40% after the first year of ulcer healing and 60% within three years; this increases to 65% after five years [1]. Additionally, patients with a history of DFU have a 2.5 times higher risk of death than those without a history of DFU [2] with mortality rates increasing to 70% within five years of undergoing an amputation [3].

Peripheral arterial disease (PAD) is a risk factor in the development of DFU and is associated with delayed healing, increased risk of infection, and, as a consequence, subsequent amputation. Among diabetic patients with DFU, more than 50% display peripheral artery disease (PAD), which makes the DFU more difficult to heal [4].

We know that foot tissues can become ischemic due to macrovascular disease (atherosclerosis); however, when evaluating the genesis of diabetic foot complications, not only macrovascular complications but also the presence of microvascular complications seem to be important predictors for the development and the prognosis of the DFU [5]. Nowadays, the neuroischemic diabetic foot ulcer profile has become the most common type of ulcer among patients with diabetes [1]. For this reason, accurate diagnosis of PAD in patients with DFU is a critical and challenging aspect of evaluation, as it can inform decisions regarding ulcer prevention strategies and revascularization treatments.

In recent years, the implementation of endovascular revascularizations, guided by angiosomes whenever possible, has demonstrated positive effects on ulcer healing and limb-saving therapy in patients with chronic limb-threatening ischemia [6] while substantially saving healthcare costs associated with lower extremity amputations and improving the prognosis for healing.

The ability to predict DFU healing in patients with PAD has been the objective of several studies that have evaluated the usefulness of noninvasive tests [7,8,9]. However, the clinical utility of performing prognostic testing in patients with DFU remains uncertain. A recent study showed that no tests, including the ankle brachial pressure index (ABPI), ankle pressure (AP), the toe brachial pressure index (TBPI), toe pressure (TP), or transcutaneous oxygen pressure (TcPO_2_), worked well enough to be used in isolation as a prognostic marker for predicting a cure of DFU [10]. Moreover, it is more difficult to establish a prognostic factor for healing based on the changes in these tests after endovascular surgery.

Our study aim was to analyze the best noninvasive tests prognosis marker in patients with DFU who underwent endovascular revascularization based on clinical outcomes, such as the rate of healing, time to heal, and free amputation survival after at least a six-month follow-up period.

## 2. Methods

### 2.1. Study Design

A multicentric prospective observational study was performed with 28 participants with ischemic or neuroischemic DFU who came to the participant centers and underwent endovascular revascularization between January 2022 and March 2023. This study protocol received full approval from the Ethics Committee of the Hospital Clínico San Carlos (21/596-E).

### 2.2. Participants

The inclusion criteria included patients with diabetes type 1 or 2 over the age of 18 with the presence of ischemic or neuroischemic DFU, which required revascularization. Furthermore, wound stages, based on the University of Texas wound system classification [11], had to be rated IC, IIC, IIIC, ID, IID, or IIID. Additional measurements were as follows: the ankle brachial index pressure < 0.4, ankle pressure < 50 mmHg, toe pressure < 30 mmHg, or TcPO_2_ < 30 mmHg [12].

Patients were excluded from the study if they had acute Charcot neuroarthropathy, neuropathic diabetic foot, the presence of lymphedema, amputation of the contralateral limb, DFU that made it impossible to measure noninvasive vascular tests, inability to walk autonomously, life expectancy of less than six months, malignancy treatment patients, unfeasible lower limb, or physical or psychological incapacity to participate in the study.

### 2.3. Procedures

All patients who consecutively and voluntarily met the inclusion criteria were included in the study after signing the informed consent at one of the following research centers:

Diabetic Foot Unit. Podiatric Clinic, Complutense University of Madrid, Spain.

Vascular Surgery Department, Hospital Fundación Alcorcón, Madrid, Spain.

Vascular Surgery Department, Hospital Universitario Ramón y Cajal, Madrid, Spain.

Vascular Surgery Department, Hospital Quirón Pozuelo, Madrid, Spain.

Vascular Surgery Department, Hospital Quirón La Luz, Madrid, Spain.

After the inclusion and before endovascular revascularization, all participants were referred to the Diabetic Foot Unit, Podiatry Clinic at University Complutense of Madrid, where noninvasive tests were performed using the PeriFlux 6000 System, Perimed, Sweden (Visit 0).

Sensorimotor neuropathy of DFUs was diagnosed using a biotensiometer (both from Novalab Iberica, Madrid, Spain) and Semmes–Weinstein 5.07/10 g monofilament. Patients who could not feel one of the two tests were diagnosed with neuropathy [13].

Patients were followed for at least six months after revascularization, and noninvasive tests were performed again four weeks after revascularization (Visit 1).

### 2.4. Evaluation of Noninvasive Tests at Visit 0 and Visit 1

Patients were placed in a supine position for at least ten minutes before starting the measurements in a heated room (20–23 °C). Toe systolic pressure (TP), ankle systolic pressure (AP), the ankle brachial pressure index (ABPI), the toe brachial pressure index (TBPI), transcutaneous pressure of oxygen (TcPO_2_), and skin perfusion pressure (SPP), were evaluated using PeriFlux 6000 System, Perimed, Sweden. Brachial artery pressures from both arms were evaluated, and the highest reading was used to calculate ABPI and TBPI. Three measurements were made for each test and a mean was estimated. All tests were performed by two clinicians experienced in the use of the PeriFlux 6000 System, Perimed, Sweden.

All patients received dry dressings to control local infection signs before the revascularization procedure, wet dressings to control local infection signs after the revascularization procedure, and proper offloading (a removable walker cast based on the functioning and ambulatory status of the patient), following the protocol of the participating research centers.

The management of diabetic foot osteomyelitis was performed by surgical or medical treatment. All surgeries were performed by the same surgeon, a specialist in conservative foot surgery, which is defined as procedures in which only infected bone and nonviable soft tissue are removed but no amputation of any part of the foot is undertaken [14]. Patients who were managed with medical treatment first received empirical antibiotics, following the recommendation of international guidelines [15], and the treatment was then modified according to the result of the bone culture. Antibiotic treatment was maintained for six weeks [16].

### 2.5. Endovascular Revascularization

Endovascular revascularizations were performed by a group of vascular surgeons dedicated to the treatment of this type of pathology.

All patients received preoperative antiplatelet therapy (100 mg ASA) at least three days prior to the intervention, which was continued postoperatively along with the use of statins. In those patients without prior indication for anticoagulation, dual antiplatelet therapy with Clopidogrel 75 mg was prescribed for at least three to six months.

For the treatment of lesions in the femoropopliteal segment, plain old balloon angioplasty (POBA), drug-coated balloons (DCB), or stents (bailout stent strategy) were used depending on the characteristics and length of the lesion. In the infrapopliteal territory, only POBA or DCB were used. 

Selections for direct revascularization or indirect revascularization were performed before the intervention based on preoperative evaluation. When the ulcer was located at the dorsum of the foot, revascularization of the anterior tibial artery was preferred. Ulcers or gangrenous lesions located on the plantar surface of the foot were prioritized for treatment of the posterior tibial artery. For ulcers located on the lateral aspect of the heel or external malleolus, efforts were focused on opening the peroneal artery. If the direct artery of the lesion was not revascularized, treatment of the tibial vessel that best connects with the plantar arch was attempted.

For patients with combined infection, the appropriate antibiotics were selected perioperatively according to the culture results.

### 2.6. Sample Size

Sample size calculation was performed using R-project version 3.6.2 (per package). It was determined, based on a desired power of 80% with a β level of 20%, with an α level of 0.05 and a confidence interval of 95%, to use logistic regression models with the independent variables analyzed for this study. At least 24 participants were included in the study.

### 2.7. Statistical Analysis

All statistical analyses were performed using SPSS statistics version 25.0 for Mac OS (SPSS Inc., Chicago, IL, USA, EE. UU). Qualitative variables were presented as percentages and frequencies, while quantitative variables were presented as means and standard deviations (SDs) except for those variables related to time, which were presented as medians and interquartile ranges (IQRs). The assumption of normality of all continuous variables was verified using the Kolmogorov–Smirnov test, for which the normally distributed variables were *p* ≥ 0.05).

Student’s *t*-test was performed to compare quantitative variables. In the case of non-normally distributed variables, the Wilcoxon test for related samples or the Wilcoxon–Mann Whitney for independent samples was performed. To identify differences in qualitative variables, the chi-square test was used. To select the optimal diagnostic cut-off points of the noninvasive tests in predicting a healing ulcer, ROC curves were employed. In addition, the sensitivity, specificity, positive predictive value (PPV), negative predictive value (NPV), positive likelihood ratio (PLR), and negative likelihood ratio (NLR) were calculated for all the tests.

Additionally, to analyze the proportion of patients with wound-healing achievement and the appearance of complications, Kaplan–Meier curves were used during the six-month follow-up period. We identified independent predictors for wound healing and major amputation using a logistic regression model with wound healing or major amputation as the dependent variable and the list of independent variables consisted of AP, TP, ABPI, TBPI, TcPO_2_, and SPP. Covariates with a *p*-value of less than 0.01 in the univariate analyses were included in the model. *p*-values of < 0.05 were considered statistically significant with a confidence interval of 95% (α of 5%).

### 2.8. Outcome Measures

The primary clinical outcome was an evaluation of the clinical evolution of noninvasive tests comparing Visit 0 (before endovascular revascularization) and Visit 1 (four weeks after endovascular revascularization), estimating the sensitivity for predicting wound healing of noninvasive tests at six months following initial recruitment. Wound healing was considered achieved when the patient presented a total epithelization of the DFU confirmed at least two weeks after wound closure. Time to heal was defined as the time from the inclusion day of the ulcer in the study to wound healing, measured in weeks.

The secondary outcome measure was to determine the best combination of noninvasive diagnostic tests for predicting wound healing.

The third outcome measure was to estimate the rates of major amputation and mortality of the study population, estimating the sensitivity for predicting lower-extremity survival of noninvasive tests at six months following initial recruitment.

Furthermore, the pain intensity of patients was valued at Visit 0 and Visit 1 through a numerical pain score from zero to ten, where zero indicated the absence of pain and ten represented maximum pain [17].

## 3. Results

Between January 2022 and March 2023, a total of 53 participants were assessed by noninvasive testing using the Periflux 6000 system. Twenty-five patients were excluded from the study for the following reasons: fourteen patients were lost to follow-up; revascularization was not possible for seven patients; two patients required bypass revascularization; and two patients refused revascularization. Finally, 28 patients with ischemic or neuroischemic DFU were included in the study and underwent an endovascular revascularization. Therefore, these patients were included in the follow-up study.

Patients’ demographic characteristics, DM, related foot complications, and vascular status at Visit 0, stratified by healing at six months following recruitment, are included in Table 1.

During the six-month follow-up study, 71.43% (n = 20) of DFUs healed, four patients (14.3%) received major amputations, and one patient (3.5%) died. The medium healing time in our study population was 12 weeks (6.5–28.50 weeks). Additionally, eleven patients (39.3%) received conservative surgery, of which nine patients healed during follow-up and two did not. The medium pain intensity in our study population was 5 (0.0–7.75) at Visit 0 (before revascularization) and 0.0 (0.0–3.5) at Visit 1(after revascularization), *p* = 0.002.

Table 2 depicts values of noninvasive tests comparing Visit 0 and Visit 1 (four weeks after revascularization).

Table 3 depicts the values of noninvasive tests at Visit 1 (four weeks after revascularization) comparing healed and non-healed patients during a follow-up of six months.

Variables with a *p*-value of less than 0.01 in the univariate analyses (TP and TcPO_2_) were included in the logistic regression analyses. TcPO_2_ measurement was identified as an independent noninvasive vascular test to predict wound healing at six months (*p* = 0.042; 95% CI, 1.05–1.32).

**Figure 1:**
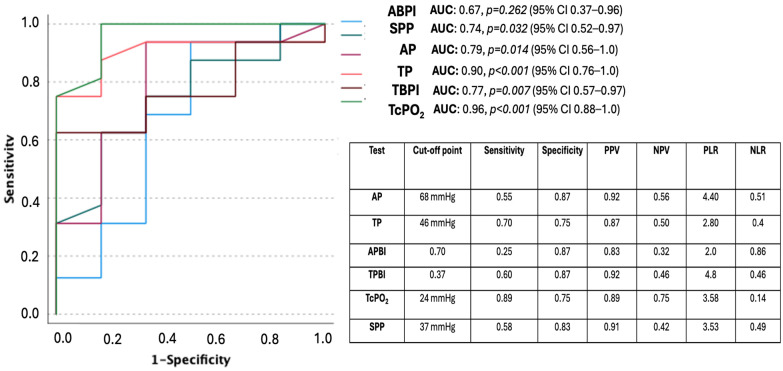
ROC curves for noninvasive tests based on ulcer healing and optimal prognosis cut-off points based on sensitivity, specificity, positive predictive value (PPV), negative predictive value (NPV), positive likelihood ratio (PLR), and negative likelihood ratio (NLR). **Abbreviations**: AP, ankle pressure; TP, toe pressure; ABPI, ankle brachial pressure index; TBPI, toe brachial pressure index; TcPO_2_ transcutaneous oxygen pressure; SPP, skin perfusion pressure. *p* < 0.05 indicates statistical significance.

Figure 1 presents the ROC curves for noninvasive tests based on ulcer healing with the optimal prognosis cut-off points based on sensitivity, specificity, positive predictive value (PPV), negative predictive value (NPV), positive likelihood ratio (PLR), and negative likelihood ratio (NLR). 

Table 4 presents the prognostic value of wound healing of diagnostic combinations based on the cut-off points previously established for noninvasive tests.

**Figure 2:**
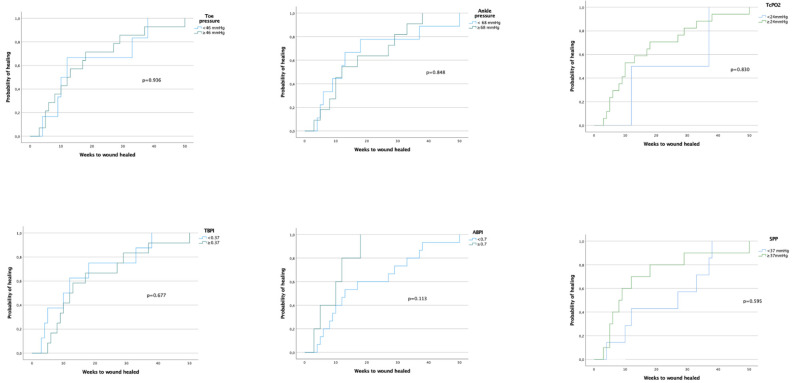
Kaplan–Meier survival curves of time to wound healing for noninvasive tests using the optimal cut-off point for each test.

Figure 2 shows the Kaplan–Meier curves of noninvasive tests using the optimal cut-off point of healing for each test.

We did not find statistically significant differences in the median healing times between patients who also underwent conservative surgery for the treatment of diabetic foot osteomyelitis (ten weeks (7.0–12.9)) versus those who did not require conservative surgery (17.0 weeks (7.3–26.7)), *p* = 0.406.

Table 5 depicts values of noninvasive tests at Visit 1 (four weeks after revascularization) comparing patients who received or did not receive major amputation during the follow-up of six months.

Variables with a *p*-value of less than 0.01 in the univariate analyses (TcPO_2_) were included in the logistic regression analyses. TcpO_2_ measurement was not identified as an independent noninvasive vascular test to predict major amputation at six months (*p* = 0.063; 95% CI, 0.68–1.01).

**Figure 3:**
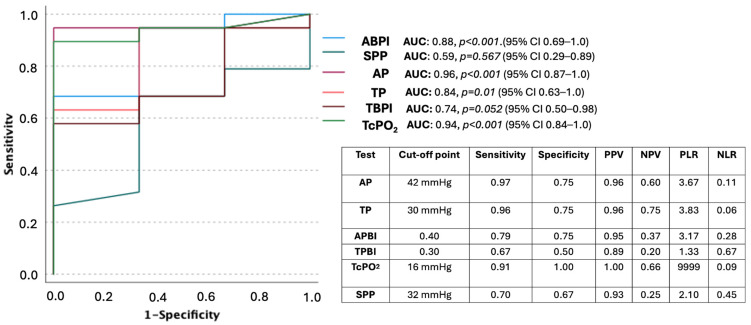
ROC curves for noninvasive tests based on not suffering a major amputation and optimal prognosis cut-off points for sensitivity, specificity, positive predictive value (PPV), negative predictive value (NPV), positive likelihood ratio (PLR), and negative likelihood ratio (NLR). **Abbreviations**: AP, ankle pressure; TP, toe pressure; ABPI, ankle brachial pressure index; TBPI, toe brachial pressure index; TcPO_2_ transcutaneous oxygen pressure; SPP, skin perfusion pressure. *p* < 0.05 indicates statistical significance.

Figure 3 presents ROC curves for noninvasive tests based on not suffering a major amputation with the optimal prognosis cut-off points for sensitivity, specificity, positive predictive value (PPV), negative predictive value (NPV), positive likelihood ratio (PLR), and negative likelihood ratio (NLR).

## 4. Discussion

In this multicenter study, we observed statistically significant increases in all noninvasive tests four weeks after revascularization (Visit 1) in our study population (Table 2). Moreover, when we compared values of noninvasive tests at Visit 1 stratifying healed and not healed patients during follow-up, we observed a statistically significant increase in values of absolute systolic pressures of the ankle, toe, and transcutaneous oxygen pressure (TcPO_2_) in patients who healed (Table 3). On the other hand, the two tests that best predicted wound healing after revascularization according to the ROC curve were TcPO_2_ and absolute systolic toe pressure, with sensitivities of 0.89 and 0.70 for the cut-off points of 24 mmHg and 46 mmHg, respectively (Figure 1).

Moreover, we found that TcPO_2_ was an independent noninvasive vascular test to predict wound healing at six months. These findings concur with the study of Mateo-Moral et al. who found that TcPO_2_ values were the best predictors of ulcer healing in patients with DFU after a 24-week follow-up period with a sensitivity of 0.91 for the cut-off points of 28 mmHg [8].

We also observed that when TcPO_2_ and systolic toe pressure were combined, the prognostic value of wound healing increased with a sensitivity of 0.95 (Table 4). The intersocietal guidelines on peripheral artery disease in people with diabetes and a foot ulcer suggest performing a toe pressure measurement to assess the likelihood of healing and amputation [18]. Based on these guidelines, toe pressure ≥ 30 mmHg increases the pretest probability of healing by up to 30%, and a value < 30 mmHg increases the pretest probability of major amputation by approximately 20%. In our study, the best cut-off point for predicting wound healing using toe pressure measurement was 46 mmHg, finding a healing rate of 70% of patients with a toe pressure ≥ 46 mmHg. On the other hand, three of the four patients who received a major amputation (75%) had toe pressure < 30 mmHg. Moreover, this guideline considers that TcPO_2_ ≥ 25 mmHg increases the pretest probability of healing by up to 45%, and a value < 25 mmHg has been shown to increase the pretest probability of major amputation by approximately 20%.

In our study, the best cut-off point for predicting wound healing using TcPO_2_ measurement was 24 mmHg, finding a healing rate of 85% of patients with a TcPO_2_ ≥ 24 mmHg. On the other hand, 100% of the patients (n = 4) who suffered major amputation had a TcPO_2_ < 16 mmHg. Our results, despite TcPO_2_ measurement, did not identify independent noninvasive vascular tests to predict major amputation at six months. A large majority (91.3%) of our patients with TcPO_2_ ≥ 16 mmHg had survival of their lower extremities (Figure 3).

Moreover, we found that skin perfusion pressure could predict wound healing according to the ROC curve (Figure 1), although with a lower sensitivity (0.58) than TcPO_2_ or toe pressure in the wound-healing prognosis, for a cut-off point of 37 mmHg. Our findings are in line with a recent systematic review that determined that a toe pressure ≥ 30 mmHg, TcPO_2_ ≥ 25 mmHg, and skin perfusion pressure ≥ 40 mmHg were associated with a moderate to large increase in pretest probability of healing in people with DFUs [19]. Chuter et al. found in a systematic review that an ABI < 0.4 demonstrated the largest increase in the pretest probability of a major amputation (PLR ≥ 10); nevertheless, an ABI ≥ 0.9 did not increase the pretest probability of DFU healing [19]. These results were similar to ours, where we found that 79% of patients with ABI ≥ 0.4 did not suffer a major amputation (PLR = 3.17). Furthermore, the best cut-off point for ABI was ≥0.70 with low sensitivity in wound healing (0.25). Related to the ankle pressure threshold, we found the best cut-off point for predicting wound healing was ≥68 mmHg with a sensitivity of 0.55 and a PLR of 4.40.

Wallin et al. used a similar threshold of ankle pressure (≥70 mmHg) and reported a PLR of 3.44 indicating a 15–20% increased likelihood of DFU healing [20]. Regarding TPBI, we found the best threshold of ≥ 0.37 for predicting wound healing with a sensitivity of 0.60, but no significant differences were found in lower-extremity survival using ROC curves for this test (Figure 3). In this regard, Chuter et al. suggest that TPBI is of limited use as a prognostic marker of either healing or amputation outcomes and should not be used as a primary test for this purpose because the value of TPBI will be affected by the magnitude of the brachial systolic pressure [19].

Our findings highlight the role of microcirculation in the healing of patients with diabetic foot ulcers who underwent revascularization and the importance of combining microcirculation and macrocirculation measurement tests to establish wound-healing and lower-extremity-survival prognoses in this population. This concept is in line with the study of Ferraresi et al., which demonstrates that critical limb ischemia in patients with diabetes is often associated with small artery disease and the failure of the distribution system of the foot, compromising the transmission system of blood to the foot [21].

The healing rate in our study population was 71.43% (n = 20) with a medium healing time of 12 weeks. When we evaluated healing times using Kaplan–Meier curves based on the cut-off points of each test (Figure 2), we did not observe statistically significant differences, suggesting that these tests predict healing but not healing times. We must consider that other variables may influence healing times, namely the presence and type of microorganisms involved in the infection, poor glycemic control, high blood pressure, blood parameters, the size of the DFU, history of amputation, and location of the DFU [22,23,24,25]. To our knowledge, this is the first study to evaluate the clinical evolution of noninvasive tests in patients who have undergone an endovascular revascularization process, highlighting the importance of the evaluation of microcirculation in the healing prognosis in patients with DFU.

One strength of this study is that the study population was followed for six months after revascularization. However, there are also limitations. The first is the small sample size and the second is that stratification was not performed on the type of endovascular surgery (direct, indirect, angiosome-guided). Therefore, future studies are suggested to expand the sample size, follow-up time, and type of endovascular surgery.

## 5. Conclusions

TcPO_2_ and absolute systolic toe pressure were the two tests that best predicted wound healing in patients who underwent endovascular revascularization. Moreover, TcPO_2_ was an independent noninvasive vascular test used to predict wound healing at six months. Our findings highlight the role of microcirculation in the healing of patients with diabetic foot ulcers who underwent revascularization and the importance of combining microcirculation and macrocirculation measurement tests to establish wound-healing and lower-extremity-survival prognoses in this population.

## Figures and Tables

**Table 1:** Demographics characteristics of the study population.

Variables	Not Healed (n = 8)	Healed (n = 20)	*p*-Value
Male, n (%)	7 (87.5)	17 (85)	0.864
Female, n (%)	1 (12.5)	3 (15)	
Age (years), mean ± SD	76.75 ± 13.23	75.15 ± 9.03	0.047 *
Type 1 DM, n (%)	0 (0)	1 (5)	0.520
Type 2 DM, n (%)	8 (100)	19 (95)	
DM duration (years)	19.25 ± 9.92	24.15 ± 10.95	0.670
HbA1c (%)	7.0 (6.5–7.9)	7.2 (6.6–8.8)	0.494
BMI, mean ± SD	22.51 ± 1.29	24.81 ± 2.98	0.057
Hypertension, n (%)	7 (87.5)	20 (100)	0.107
Hypercholesterolemia, n (%)	8 (100)	15 (75)	0.119
Neuropathy, n (%)	6 (75)	11 (55)	0.328
Retinopathy, n (%)	3 37.5)	4 (20)	0.334
Nephropathy, n (%)	2 (25)	2 (10)	0.306
Cardiopathy, n (%)	7 (87.5)	11 (55)	0.105
Previous amputation, n (%)	2 (25)	5 (25)	1.000
Texas grade and stage (%)			
1C	4 (50)	10 (50)	1.000
2C	1 (12.5)	0 (0)	0.107
3C	0 (0)	1 (5)	0.520
1D	1 (12.5)	1 (5)	0.486
2D	2 (25)	1 (5)	0.122
3D	0 (0)	7 (33.3)	0.053
Presence of distal pulses, n (%)	0 (0)	0 (0)	NA
AP	52.5 (21.25; 99.75)	42 (13.0; 72.25)	0.636
TP	25.0 (17.0; 50.0)	26.0 (17.5; 40.5)	0.940
ABPI	0.42 (0.16; 0.70)	0.32 (0.09; 0.52)	0.381
TBPI	0.19 (0.11; 0.35)	0.19 (0.11; 0.30)	0.862
TcPO_2_ (mmHg)	23.25 ± 20.07	35.40 ± 22.3	0.193
SPP (mmHg)	36.5 (20.25; 73.5)	38.5 (16.75; 54.0)	0.820
RAC, n (%)	4 (57.1)	17 (81)	0.204
Antiplatelet treatments, n (%)	5 (71.4)	18 (85.7)	0.393
Statins, n (%)	7 (100)	17 (81)	0.212
Anticoagulant, n (%)	3 (42.9)	5 (23.8)	0.334
Antihypertensives, n (%)	6 (85.7)	21 (100)	0.078
Oral antidiabetics, n (%)	5 (71.4)	16 (76.2)	0.801
Insulin, n (%)	3 (42.9)	9 (42.9)	1.000

Mean ± standard deviation or median (25th, 75th percentile), as appropriate. **Abbreviations**: SD, standard deviation; DM, diabetes mellitus; Glycated hemoglobin, HbA1c; BMI, body mass index; AP, ankle pressure; TP, toe pressure; ABPI, ankle brachial pressure index; TBPI, toe brachial pressure index; TcPO_2_, transcutaneous oxygen pressure; SPP, skin perfusion pressure; NA, not available. * *p* < 0.05.

**Table 2:** Values of noninvasive test comparing Visit 0 and Visit 1.

	AP	TP	ABPI	TBPI	TcPO_2_	SPP
Visit 0 (Before revascularization)	48.5 (17.0; 72.75)	26.0 (17.5; 40.5)	0.32 (0.13; 0.56)	0.19 (0.11; 0.30)	33.5 (11.0; 49.0)	35.0 (29.0; 69.0)
Visit 1 (4 weeks after revascularization)	65.5 (46.0; 63.75)	49.0 (34.2; 63.75)	0.51 (0.38; 0.67)	0.34 (0.25; 0.43)	39.0 (19; 59.0)	38.5 (20.25; 59.5)
*p*-value	*p* < 0.001 *	*p* < 0.001 *	*p* = 0.002 *	*p* < 0.001 *	*p* < 0.001 *	*p* = 0.013 *

**Abbreviations**: AP, ankle pressure; TP, toe pressure; ABPI, ankle brachial pressure index; TBPI, toe brachial pressure index; TcPO_2_ transcutaneous oxygen pressure; SPP, skin perfusion pressure. * *p* < 0.05.

**Table 3:** Values of noninvasive tests at Visit 1 comparing healed and non-healed patients during a follow-up of six months.

	AP	TP	ABPI	TBPI	TcPO_2_	SPP
Healed n = 20	85.20 ± 37.5	55.0 (17.5; 40.5)	0.57 ± 0.22	0.34 (0.27; 0.45)	49.05 ± 18.5	55.76 ± 34.11
Not healed n = 8	46.75 ± 28.09	33.0 (27.25; 43.25)	0.39 ± 0.26	0.29 (0.18; 0.35)	12.12 ± 12.33	30.66 ± 21.15
*p*-value	*p* = 0.015 *	*p* = 0.008 *	*p* = 0.077	*p* = 0.079	*p* < 0.001 *	*p* = 0.108

Normally distributed variables were represented as mean ± standard deviations and non-normally distributed variables were represented as median and interquartile range (1st quartile, 3rd quartile). **Abbreviations**: AP, ankle pressure; TP, toe pressure; ABPI, ankle brachial pressure index; TBPI, toe brachial pressure index; TcPO_2_ transcutaneous oxygen pressure; SPP, skin perfusion pressure. * *p* < 0.05.

**Table 4:** Prognostic value of wound healing of diagnostic combinations based on the cut-off points previously established for noninvasive tests.

Combination of Noninvasive Tests	Cut-Off Point	Sensitivity	Specificity	PPV	NPV	PLR	NLR
AP and TP	68–46 mmHg	0.85	0.62	0.85	0.62	2.27	0.24
ABPI and TBPI	0.70–0.37	0.70	0.75	0.87	0.50	2.8	0.40
TcPO_2_ and SPP	24–37 mm Hg	0.90	0.62	0.96	0.29	2.4	0.16
TP and TcPO_2_	46–24 mmHg	0.95	0.62	0.86	0.83	2.53	0.08

**Abbreviations**: AP, ankle pressure; TP, toe pressure; ABPI, ankle brachial pressure index; TBPI, toe brachial pressure index; TcPO_2_ transcutaneous oxygen pressure; SPP, skin perfusion pressure; PPV, positive predictive value; NPV; negative predictive value; PLR, positive likelihood ratio; NLR, negative likelihood ratio.

**Table 5:** Values of noninvasive tests at Visit 1 comparing patients who received or did not receive major amputation during the follow-up of six months.

	AP	TP	ABPI	TBPI	TcPO_2_	SPP
Major amputation n = 4	30.75 ± 15.33	27.5 (15.75; 41.5)	0.25 ± 0.16	0.23 (0.10; 0.32)	6.00 ± 5.48	40.66 ± 24.95
Not Major amputation n = 24	81.46 ± 36.81	52.0 (35.0; 69.5)	0.56 ± 0.22	0.38 (0.27; 0.45)	43.69 ± 21.27	50.50 ± 34.14
*p*-value	*p* = 0.013 *	*p* = 0.012 *	*p* = 0.013 *	*p* = 0.059	*p* < 0.001 *	*p* = 0.639

**Abbreviations**: AP, ankle pressure; TP, toe pressure; ABPI, ankle brachial pressure index; TBPI, toe brachial pressure index; TcPO_2_ transcutaneous oxygen pressure; SPP, skin perfusion pressure; * *p* < 0.05.

## Data Availability

The data presented in this study are available on request from the corresponding author due to ethical reasons and privacy.
